# Ultrasound as Green Technology for the Valorization of Pumpkin Leaves: Intensification of Protein Recovery

**DOI:** 10.3390/molecules29174027

**Published:** 2024-08-26

**Authors:** Jelena Mijalković, Nataša Šekuljica, Sonja Jakovetić Tanasković, Predrag Petrović, Bojana Balanč, Marija Korićanac, Ana Conić, Jelena Bakrač, Verica Đorđević, Branko Bugarski, Zorica Knežević-Jugović

**Affiliations:** 1 Department of Biochemical Engineering and Biotechnology, Faculty of Technology and Metallurgy, University of Belgrade, Karnegijeva 4, 11000 Belgrade, Serbia; jjovanovic@tmf.bg.ac.rs (J.M.); sjakovetic@tmf.bg.ac.rs (S.J.T.); mkoricanac@tmf.bg.ac.rs (M.K.); aconic.tmf@gmail.com (A.C.); 2 Innovation Centre of the Faculty of Technology and Metallurgy Ltd., Karnegijeva 4, 11000 Belgrade, Serbia; nsekuljica@tmf.bg.ac.rs (N.Š.); ppetrovic@tmf.bg.ac.rs (P.P.); bisailovic@tmf.bg.ac.rs (B.B.); jbakrac@tmf.bg.ac.rs (J.B.); 3 Department of Chemical Engineering, Faculty of Technology and Metallurgy, University of Belgrade, Karnegijeva 4, 11000 Belgrade, Serbia; vmanojlovic@tmf.bg.ac.rs (V.Đ.); branko@tmf.bg.ac.rs (B.B.)

**Keywords:** pumpkin leaves, RuBisCO-rich protein fraction, high-intensity ultrasound, protein extraction, functionality, bioactivity, protein structure

## Abstract

The recovery of valuable nutritional compounds, like proteins, from waste streams and by-products is a key strategy for enhancing production sustainability and opening up new market potential. This research aimed to use high-intensity ultrasound as an innovative technique to extract the soluble proteins from the pumpkin leaves. The impact of various sonication amplitudes and duration periods on protein yield, functional properties, antioxidant qualities, and structural characteristics, were studied. Utilization of ultrasound technology significantly increased the yield of pumpkin leaf protein by up to 40%—six times higher than maceration. The ultrasound extraction provided a RuBisCO-rich protein fraction with high radical scavenging and chelating activities, especially at 40% amplitude. Cavitation modified the tertiary and secondary structures of leaf proteins: the amount of *α*-helix changed based on amplitude (12.3–37.7%), the amount of random coil increased to 20.4%, and the amount of *β*-turn reduced from 31 to 18.6%. The alteration of the protein fluorescence spectrum (blue shift in spectrum) provides further evidence that ultrasound alters the proteins’ molecular structure in comparation with maceration; the maximum tryptophan fluorescence intensity decreased from 22.000 to 17.096. The hydrophobicity values of 76.8–101.5 were substantially higher than the maceration value of 53.4, indicating that ultrasound improved the hydrophobicity of protein surfaces. Ultrasound resulted in a significant increase in solubility in an acidic environment with the increase in sonication amplitude. A 2.4-fold increase in solubility at pH 2 becomes apparent (20% amplitude; 43.1%) versus maceration (18.2%). The emulsifying ability decreases from 6.62 to 5.13 m^2^/g once the sonication amplitude increases by 20–70%. By combining the ultrasound periods and amplitudes, it is possible to create high-value protein leaf extracts with improved properties which can find real application as food additives and dietary supplements.

## 1. Introduction

Over the last few decades, there have been several issues facing the world as a result of the expanding human population and the sharply rising demand for goods derived from animals [1,2]. According to estimates, 20% of greenhouse gas emissions that contribute to climate change come from animal husbandry. Other effects include the loss of evergreen forests and arable land, the rapid extinction of species and biodiversity, and an increase in land, air, and water pollution worldwide. Among the principal strategies for tackling the excessive use of animals for human nutrition is to create sustainable protein sources with significantly less of an impact on the environment. The most widely available plant-based proteins, which are nonetheless among the least utilized, are green leaf proteins [3,4,5,6]. Protein content in green leaves often varies greatly amongst plant species, ranging from 5 to 35% on a dry basis. These proteins can be categorized into two groups: the water-soluble proteins (mostly cytoplasmic) that make up the white fraction after extraction, and the water-insoluble proteins (primarily membrane proteins, like thylakoids) that comprise the majority of those within the green fraction [5,7,8]. RuBisCO (ribulose-1,5-bisphosphate carboxylase/oxygenase; E.C. 4.1.1.39) is the primary protein found in most green leaves; it accounts for 50–65% of soluble proteins and ~2.5% of the global mass of leaves. Furthermore, RuBisCO mediates the first and key reaction of photosynthetic CO_2_ assimilation. However, RuBisCO has a very low turnover rate and specificity towards CO_2_, and as such, it is proclaimed to be the most abundant protein in the world. RuBisCO originates in four forms, but form I, which has a molecular weight of about 560 kDa, is the one that is primarily found in plant leaves, and it consists of eight large (50–55 kDa) and eight small (12–15 kDa) subunits [4,8,9]. RuBisCO, a high-nutrient food protein, meets WHO standards and has a high content of sulfur-containing amino acids, and phenylalanine, leucine, tyrosine, and tryptophan [4,10].

In the unrefined oil industry, virgin pumpkin oil represents a seed oil which is widely used in cooking and cosmetics and which is rich in vitamins and omega fatty acids necessary for normal body function. This is why, in fields around the world, special attention is being paid to the cultivation of oil pumpkin (*Cucurbita pepo* L.). According to the Food and Agriculture Organization (FAO), worldwide pumpkin production reached a high level of 24,461,368 tons in 2020. It has been forecasted that the global pumpkin seed market is poised to grow by USD 904.2 million from 2022 to 2026 [11]. As the use of pumpkin seeds increases, more green leaves remain after processing; this biomass may be a huge source of RuBisCO proteins. Using this green biomass is generally advantageous from an economic and environmental standpoint and it aligns with the zero-waste concept [5,6].

Leaf source, extraction method, and product functionality all need to be taken into account when thinking about leaf utilization. When selecting a leaf source, one must consider certain factors, like protein content, availability (location, season), and future applications. In addition, it is important to assess how the process will affect the environment in order to reduce waste, water usage, and pollution [2,12]. Theoretically, it is possible to separate proteins extraction technology into two categories: conventional and non-conventional (green). Due to their cost and ease of use, conventional procedures including grinding, pressing, and selective precipitation were originally established. The conventional procedures frequently end with protein constituents that may be aggregated or denatured, resulting in elevated levels of contaminants, as well as in a low extraction yield [1,12,13,14]. Therefore, the utilization of green extraction technology for the valorization of agro-waste streams is being explored, such as using high-intensity ultrasound, high-voltage electric fields, high-pressure, microwaves, cold plasma, and hydrolytic enzymes. By leveraging the aforementioned technologies, the yield and the nutritional value of extracted proteins have been improved while extraction time, energy and solvent consumption are reduced [15,16].

As an alternative, ultrasound technology is a straightforward and efficient green physical method that uses high-intensity (often in the range of 10–1000 W/m^2^) and low-frequency waves (16–100 kHz) to modify the molecular structures of proteins, improving their physicochemical properties, and expanding their range of applications. The main advantages associated with ultrasound technology include its rapid processing time, simplicity of manual operation, and inexpensive maintenance [5,14,17]. In a liquid system, high-intensity ultrasound induces a series of compression and refraction waves in the liquid medium’s molecules. This triggers small bubbles to develop, expand, and violently collapse. The hot spot, which has temperatures between 4500–5000 K and pressures above 1000 atm, releases a significant quantity of highly localized energy [18]. In precise terms, rapid pressure changes induce cavitation bubbles to expand and collapse near the surfaces of plant materials. These bubbles generate shockwaves which damage plant cell walls. Therefore, the solvent is able to penetrate into the cells more easily, thereby promoting the solubilization of the intracellular proteins through the better exposure of hydrophilic groups of amino acids to the solvent [19,20].

Ultrasound can trigger chemical reactions by creating and dissipating small gas bubbles during a procedure. There are three distinct areas where chemical interactions occur: (1) in the high-temperature region of a collapsing gas bubble; (2) in the region where the surrounding liquid and hot gas phase meet at the interfacial; and (3) in the bulk of the solution where solutes are reacted with by free radicals that were generated in the cavitation bubbles but were not scavenged in the interfacial region [20,21,22]. Some of the most important physical effects of cavitation are shock waves, microcurrents, microjets, turbulence, and shear forces that modify the protein structure through the decomposition of intermolecular interaction forces, such as hydrogen bonds, disulfide bridges, hydrophobic interactions, electrostatic forces, and Van der Waals interactions [18]. The characteristics of several leaf proteins are considerably impacted by ultrasound treatment, as recent investigations have shown. Hadidi et al. [23] used an ultrasonic ultrafiltration-assisted alkaline isoelectric precipitation technique to isolate proteins from alfalfa leaves with an improved extraction rate in comparison with conventional alkaline-isoelectric precipitation. Ultrasound-assisted extraction (UAE) proved to be a better alternative method for total soluble protein extraction from sugar beet leaves [10] and mulberry leaves [24], and was able to provide high yield of proteins with enhanced structural and functional properties.

To date, RuBisCO is believed to be a promising protein source for food products. To the best of our knowledge, the oil pumpkin side streams, green leaves, have not previously been investigated as alternative RuBisCO protein sources. Similarly, there is a lack of research on the potential applications of ultrasound technology for protein extraction from pumpkin leaves. Therefore, this study aimed to investigate different ultrasound parameters, including duration time and amplitudes, on protein yield, and the physicochemical, functional properties, biological activities, and secondary and tertiary structures of isolated proteins. This study provides a detailed review of the major steps and key parameters of the ultrasound extraction process, facilitating the selection of the right conditions for making sustainable food proteins out of pumpkin leaves.

## 2. Results and Discussion

### 2.1. Impact of Ultrasound-Assisted Extraction on Protein Recovery and Bioactivity of RuBisCO-Rich Protein Fractions

Extraction kinetic models have received a lot of attention in the literature because they are a simple approach to illustrate the extraction practicality of different extraction techniques. The current state of extraction kinetics is a consequence of the “green chemistry” idea, resulting in the shift towards a time–yield–efficiency orientation [25]. Here, in order to comprehend the effects of sonication on the mass transfer of soluble proteins, a non-steady state diffusion model based on Fick’s second law was taken from the literature [17,26] and used to describe the impact of ultrasound-assisted extraction time and amplitude on the protein yield, as follows:

(1) $$\frac{c_{t}-c_{0}}{c_{\infty }-c_{0}}=1-e^{-kt}$$

where *c*_∞_, *c*_0_, *k*, and *t* denote the final and initial protein contents, extraction rate (min^−1^), and ultrasound extraction time, respectively.

**Figure 1:**
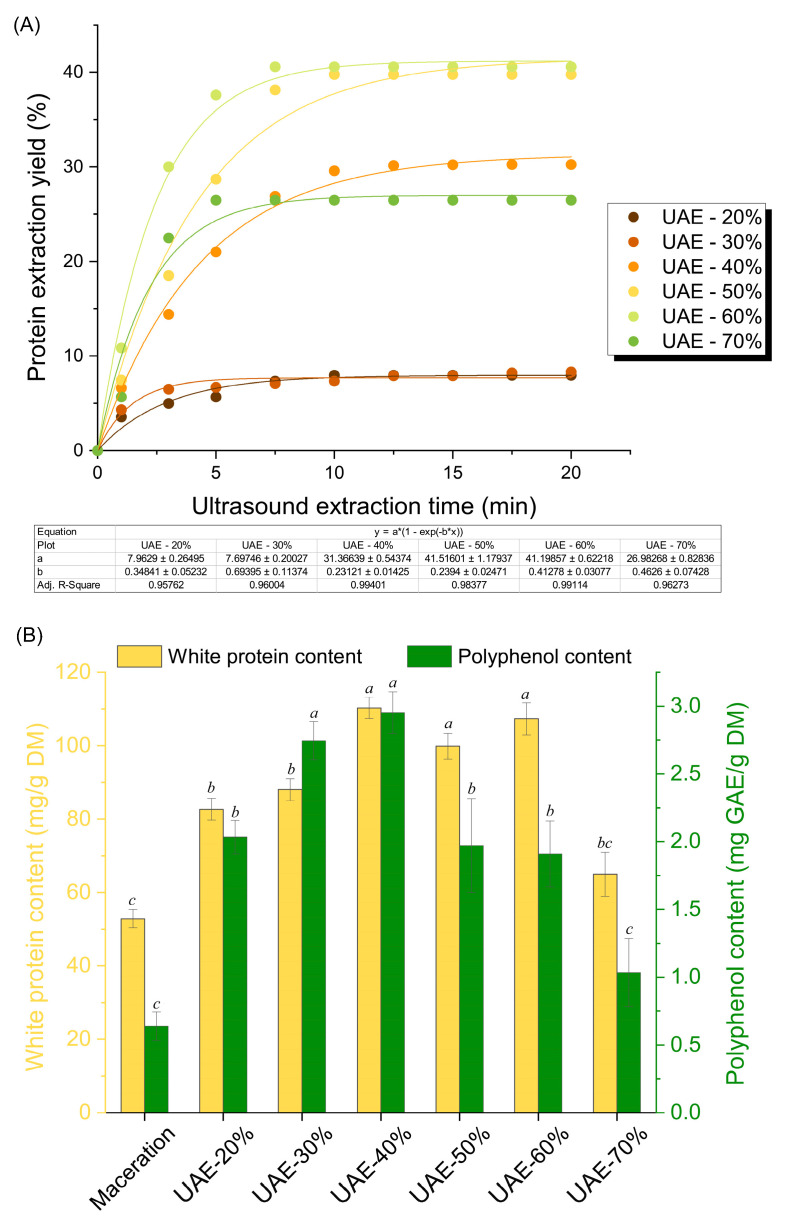
Extraction kinetics of pumpkin leaf proteins under the various ultrasound duration times and amplitudes (**A**). The impact of ultrasound amplitudes on the content of white proteins and polyphenols, extracted after 10 min of ultrasound-assisted processing (**B**). Means with different letters for the same bar groups are significantly different (*p* < 0.05). Legend: UAE-20%, UAE-30%, UAE-40%, UAE-50%, UAE-60%, and UAE-70% mean an ultrasound-assisted extraction process at 20, 30, 40, 50, 60, and 70% amplitudes, respectively.

The ultrasound probe used in this investigation produced a low-frequency, high-intensity ultrasound at a frequency of 20 kHz, and the probe operated independently of temperature. The results presented in Figure 1 demonstrate that the soluble protein yield was significantly impacted by the studied duration time and amplitudes. As shown in Figure 1A, the protein extraction yield increases over time at an exponential rate, with two prominent phases in the extraction process. In the beginning, i.e., the washing stage (about 5 min), the protein contents increase drastically with time, with an extraction efficiency of over 80% of the final contents, thus significantly decreasing the diffusion time. Nonetheless, the trend of this increase reduces quickly as it enters the slower diffusion stage and achieves a plateau, which may be caused by the reduced concentration gradient between pumpkin cells and the extraction medium, since plentiful cellular compounds are extracted into the liquid phase. Ultrasound-assisted extraction provides a higher efficiency over time as it promotes the release of cellular protein much more significantly than maceration. The protein contents in maceration (Supplementary Materials: Figure S1) stay the same in the washing stage from the beginning, with no significant increase during 60 min. This promotion effect prominently acts on the washing stage considering the high increase rate of protein content rather than in the diffusion stage, where only ~20% was recovered in 15 min, revealing that a lower time can be applied to save time and energy. As a result, the protein extraction process is complete in nearly 10 min in all cases except for maceration, which makes this time optimal for extraction in our study. 

To further analyze the mechanism of the ultrasonic-assisted extraction of pumpkin leaf protein, the kinetic behavior was described using the mentioned kinetic model (Equation (1)). According to results, the values of *a* (i.e., the kinetic constant *k*) increase with increasing ultrasound amplitudes, and the values of *b* decrease. Increased ultrasound amplitudes from 20 to 60% considerably increased pumpkin leaf protein extraction; however, increasing the ultrasound amplitude to 70% caused protein aggregation and reduced protein extraction yield. The protein yields were advanced by 7.98, 8.34, 30.24, 39.75, and 40.60% at amplitudes of 20, 30, 40, 50, and 60% amplitudes, respectively. The protein yield under the best ultrasound conditions was six times higher than that of the control sample, which used maceration (40.6 vs. 6.5%), which confirms the superiority of ultrasound-assisted protein extraction technology. Ultrasound extraction provided protein yields of 10 g/100 g DM, while enzyme-assisted extraction by carbohydrase complex produced equally effective protein yields of 2.7 g/100 g DM, according to a group of authors’ investigation [27]. Zhao et al. [24] reported that multi-frequency ultrasound-assisted cellulase extraction produced the highest yield of mulberry leaf protein (30 g/100 g DM) when analyzed alongside traditional extraction, ultrasound extraction, and cellulase extraction procedures.

Figure 1B and Table 1 indicate the effect of ultrasound amplitudes on the concentration of white proteins and polyphenols, and, thus, on antioxidative activity. The polyphenol content increases three to four times (2.1–2.9 mg GAE/g DM) at ultrasound amplitudes of 20, 30, and 40% when compared to the maceration (0.7 mg GAE/g DM). The measured polyphenol concentration decreases at larger amplitudes, but it remains above the content resulting from maceration. This reduction is the consequence of the chemical effect of ultrasound on the water molecules. Specifically, cavitation breaks the water molecules into free radicals denoted as hydrogen and hydroxyl radicals. These radicals can react with one another to form hydrogen peroxide, which can contribute to the oxidation of polyphenols to quinone. Quinones form a chemical complex with proteins, which is why there was a noticeable decrease in antioxidant activity [22]. 

The produced protein fractions show very noticeable antioxidant qualities, including hydroxyl radical inhibition > scavenging activity to ABTS cation > metal-ion chelating activity (Table 1). This implies that proteins, polyphenols, and protein–polyphenol complexes use a combined hydrogen atom and a single electron transfer mechanism to participate in the neutralization of free radicals. The profile of antioxidant activity for the three methods is opposite; there is no unique dependence, but, in summary, it can be emphasized that the fractions extracted with ultrasound amplitudes of 20, 30, and 40%, up to ~50%, are more active in inhibiting the hydroxyl radical in comparison with the maceration sample, which is exclusively a consequence of the presence of proteins, i.e., reactive groups of the side chain in the protein sequence. Inhibition of the hydroxyl radical is a characteristic of the protein samples rather than the phenolic ones. Both the 30 and 40% ultrasound amplitudes qualified the good ability of divalent iron ions’ chelation. Furthermore, implementing an ultrasound-assisted extraction protocol led to a slight decrease in ABTS radical scavenging activity with an increase in amplitudes (20–70%). The following could be explained by the sensitivity and degradation of plant polyphenol compounds through cavitation, air exposure during the sonication, and the presence of free radicals [14].

In the closing statement, it is important to address how cavitation might affect the proteins’ amino acid profile. Therefore, the amount of released free amino acid has been determined by conducting a ninhydrin spectrophotometric analysis of *α*-amino group content. The measured values of Pro and Leu’s amino acid equivalents are provided in the Supplementary Materials, Table S1. The results reveal that ultrasound exposes *α*-amino groups, specifically Leu and Pro equivalents, in pumpkin leaf proteins, compared to maceration. Leu is exposed 1.5 times more, while Pro is exposed 4 times more. No significant differences were found across ultrasound amplitudes, so further research will focus on the parameter’s impact on total amino acid composition. Applied high-intensity ultrasound (20 kHz; amplitudes from 20 to 70%) increased the exposure of Leu and Pro α-amino groups, allowing them to interact with other protein functional groups. These results support our assumption that cavitation effect chemically changes the amino acids content versus maceration, indicating that the overall nutritional quality of extracted proteins has been modified. Our findings are consistent with the literature and confirm that cavitation and sonochemical effects cause protein structure changes, including Van der Waals forces and hydrogen bond breakage, and induce amino acid profile changes in extracted molecules. [19]. The authors reported that ultrasound-assisted alkaline extraction (13.5 min and 33.7% amplitude) increased the total amino acid contents of the protein extracts by 12.6% as compared to a control isolate from pea [28].

### 2.2. Structural and Morphological Characterization of RuBisCO-Rich Protein Fractions

Once it was discovered that high-intensity ultrasound has a significant impact on protein yield as well as total antioxidant capacity during the first phase of the investigation, the structural and particle characteristics of the separated RuBisCO-rich protein fractions influenced by applied amplitudes were examined.

#### 2.2.1. Zeta Potential, Particle Size Analysis, and Molecular Weight Distribution

**Figure 2:**
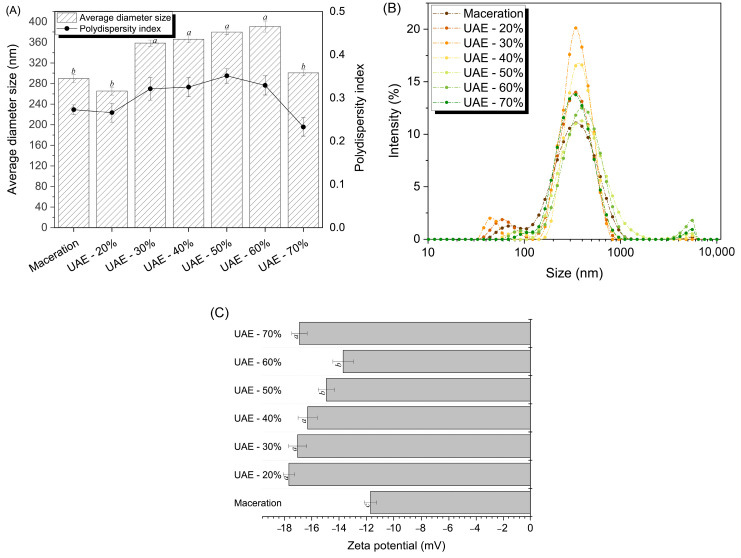
The average particle size (**A**), particle size distribution (**B**), and zeta potential (**C**) of RuBisCO-rich protein fractions extracted through ultrasound process influenced by applied amplitudes from 20 to 70%. The data are presented as the mean ± SD (*n* = 3). Means with different letters in the same subfigure (**A**,**C**) are significantly different (*p* < 0.05). Legend: UAE-20%, UAE-30%, UAE-40%, UAE-50%, UAE-60%, and UAE-70% mean ultrasound-assisted extraction process at 20, 30, 40, 50, 60, and 70% amplitudes, respectively.

Figure 2A,B, respectively, depict the size distribution and average diameter of the protein particles in the extracted RuBisCO-rich protein fractions. Pumpkin leaf protein extracts obtained by ultrasound exhibit particles with an average diameter between 265 and 390 nm. The amplitude in the range 30–60% results in larger particles (above 350 nm) compared to those produced by very low (20%; 265 nm) or very high (70%; 300 nm) amplitude or by maceration (289 nm). The explanation is that under certain amplitudes, chemical reactions occur, resulting in changes through intramolecular bonds between the smaller particles, and in the case of a very strong cavitation action, the smaller particles break up, as demonstrated by the use of a 70% amplitude. The ultrasound treatment triggered turbulence, strong shear, and a cavitation effect, which broke up some of the large insoluble proteins as evidenced by the more uniform particle distribution compared to maceration (Figure 2B). This led to fewer particle size fragments and intermolecular collisions. According to Zhao et al. [24], the average particle size produced by the ultrasound-processed (frequency 25 kHz, power density 150–450 W/L) mulberry leaf protein is substantially smaller than the average size of the particles obtained by the alkaline extraction method.

The negative zeta potential of extracted RuBisCO-rich protein fractions, which is correlated with electrostatic interactions that are indirectly related to the hydrophilicity of proteins, is shown in Figure 2C. Each fraction obtained through ultrasound displays an absolute zeta potential value significantly higher than that of the maceration sample (−11.7 mV). Under ultrasound amplitudes of 20 or 70%, the absolute potential reaches a maximum of −18 mV. The following might have occurred due to the ultrasonic mechanical forces generated, which disrupted the non-polar hydrophobic groups of pumpkin leaf proteins, exposing the hydrophobic region of the protein and promoting the negative charge on the protein surface [18,20,21,22]. This is correlated with the mean diameter of the particles, which changed by only 100 nm, not forming aggregates larger than 1000 nm for the samples obtained at 20, 30, and 40% amplitudes (Figure 2B). An increase in the absolute value of the zeta potential is associated with reduced protein aggregation and more stable protein. Vice versa is also true, which explains why samples obtained by amplitudes of 50 or 60% that have lower zeta potential values exhibit weaker repulsions between particles, as evidenced by appearance of a particle peak at 5000–6000 nm. Additionally, the application of 70% amplitude results in a decrease in the intensity of the particle peak at 5000–6000 nm and an upward trend in the zeta potential, confirming the partial disintegration of the protein aggregates.

**Figure 3:**
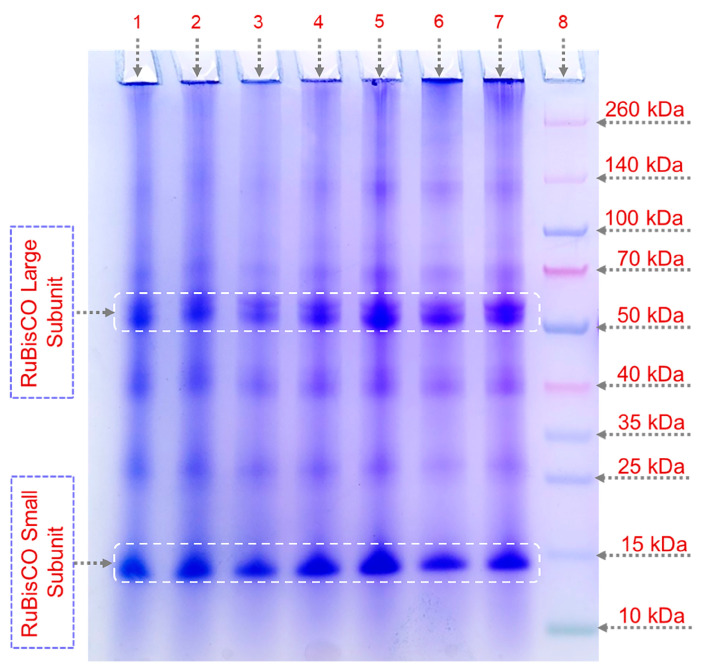
SDS-PAGE electrophoretic profiles of protein fractions extracted under the different high-intensity ultrasound amplitudes or maceration on an 8% polyacrylamide gel: line 1—maceration; line 2—UAE-20%; line 3—UAE-30%; line 4—UAE-40%; line 5—UAE-50%; line 6—UAE-60%; line 7—UAE-70%; line 8—molecular weight markers.

The molecular weight distribution of the pumpkin protein subunits is evaluated by reducing sodium dodecyl sulphate–polyacrylamide gel electrophoresis (SDS-PAGE), and the electrophoretic patterns are illustrated in Figure 3. Both large (50–55 kDa) and small (~15 kDa) RuBisCO subunits are found in each protein sample, suggesting that the applied technology is successful for the isolation of RuBisCO protein from pumpkin leaves. Comparing ultrasound-assisted and maceration extraction processes, ultrasound induces a few changes in the protein electrophoretic patterns, suggesting that the cavitation effect modifies the protein profiles at 40, 50, and 60% amplitudes through the formation of protein aggregates with molecular weights of ~100 and 70 kDa (increased band intensity) but reduces the band intensity at 25 kDa. The observed pattern coincides with the previously described particle alterations brought on by the various ultrasound wave amplitudes. The presented electropherogram indicates that ultrasound under the applied conditions did not significantly impact the RuBisCO protein subunits. In combination with the free amino acid results (Supplementary Data: Table S1), it can be concluded that the primary structure of the pumpkin leaf proteins was not affected by high-intensity ultrasound at 20 kHz. Consequently, in order to ascertain the effects of ultrasound on protein structure, additional analysis is focused on the secondary and tertiary protein structures. This finding was consistent with previous studies on plant-based proteins, especially sugar beet [10], alfalfa [8,23], and spinach [9] leaves. 

The thylakoid membrane consists of four large photosynthetic protein complexes: ATP synthase, photosystem II, cytochrome b6f, and photosystem I. Photosystems I and II are two multi-subunit pigment–protein complexes embedded in the grana and stroma of higher plants’ thylakoid membranes. These both hydrophobic proteins are trapped in the phospholipid membrane, making extraction difficult and time-consuming [29,30]. Some minor protein lines near or below 40, 30, and 25 kDa are identified; these bands appear to be the evidence for the detection of some membrane thylakoid proteins, as a result of disruption of the primary cell wall caused by the initial leaves’ lyophilization and subsequent ultrasound extraction protocol. It became apparent that in all fractions analyzed, at least four bands correspond to the expected membrane proteins, having very comparable electrophoretic mobilities. Specifically, it has been feasible to witness the subunits of the photosystem II complex, CP47 (45 kDa), CP43 (43 kDa), D1 and D2 monomer (27–30 kDa), and/or CP 29 from the photosystem I complex, whose mass is 29 kDa [29,30,31]. Only small differences in terms of protein abundance appear at amplitudes 60 and 70% with respect to maceration and other ultrasound amplitudes.

#### 2.2.2. Surface Hydrophobicity, Intrinsic Fluorescence Spectra, and Secondary Structure of the Protein Side Chain

**Figure 4:**
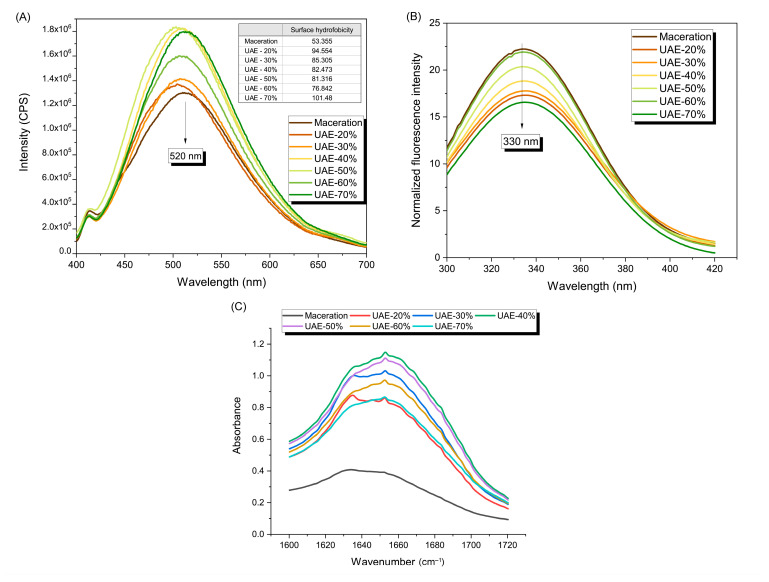
Fluorescence emission spectra and surface hydrophobicity values (**A**) and intrinsic tryptophan fluorescence spectra (**B**), which indicate tertiary structural differences between proteins extracted via maceration and ultrasound-assisted extraction influenced by different amplitudes. ATR-FTIR spectra of the characteristic amide I region (**C**) which demonstrates the changes in the secondary structure of extracted proteins. Legend: UAE-20%, UAE-30%, UAE-40%, UAE-50%, UAE-60%, and UAE-70% mean ultrasound-assisted extraction process at 20, 30, 40, 50, 60, and 70% amplitudes, respectively.

The quantity of hydrophobic groups that are exposed on the surface of a protein molecule indicates the surface hydrophobicity. This has a strong relationship with the functional properties of proteins and is frequently used to evaluate conformational changes for these proteins. Figure 4A depicts the surface hydrophobicity expressed by the pumpkin leaf protein’s relative fluorescence intensity, and calculated values *H*_0_ are given in the inserted table. The maximum fluorescence intensity of the RuBisCO-rich protein fractions peaks at 520 nm and is significantly increased with ultrasound when compared to maceration. With the exception of an amplitude of 60%, where the intensity falls in comparison to 50 and 70%, the amplitudes from 20 to 70% show an increase in fluorescence intensity at 520 nm. High-intensity ultrasound improves the hydrophobicity of protein surfaces, as evidenced by the significantly higher *H*_0_ values of the leaf proteins after ultrasound (*p* < 0.05; 94.6, 85.3, 82.5, 81.3, 76.8, and 101.5 for 20, 30, 40, 50, 60, and 70% amplitudes, respectively) as opposed to the maceration’s value (53.4). The increase in the surface hydrophobicity of the collected pumpkin leaf proteins reveals that the application of ultrasound caused the protein molecules to unfold, caused hydrophobic groups to become apparent, and caused hydrophobic amino acids that were previously hidden inside the molecules to be exposed. The complex structure of proteins has been disrupted through cavitation, prompting an outside physical field to loosen the protein structure and impair the hydrophobic networking [14,18]. Based to an interpretation of the amplitude’s behavior, increasing the amplitude from 20 to 60% causes a drop in the value of *H*_0_, the formation of a protein aggregation state, and modifications to the tertiary structure of leaf protein. By increasing the amplitude from 60 to 70%, the ultrasound probe destroys hydrophobic interactions, causing the protein structure to unfold and causing groups on the molecule’s surface to become more exposed.

We may deduce that the surface hydrophobicity has a negative correlation with the mean size of the particles while being positively correlated with the absolute value of the zeta potential by comparing the results from Figure 4A with the information gathered from Figure 2A,B. So, increases in the absolute value of zeta potential are observed to increase surface hydrophobicity (maceration vs. UAE), implying that protein molecule aggregation has been hampered by the repulsive interaction of more alike electric charges on their surface, enhancing the stability of the protein suspension while concurrently reducing the average particle size. 

Additionally, emission fluorescence spectroscopy is used to identify microenvironment changes in tryptophan residues at 330 nm [32], which are related to pumpkin protein tertiary structure modification (folding or unfolding) (Figure 4B). The maximum tryptophan fluorescence intensity decreased from 22.000 to 17.096 with the introduction of ultrasound (amplitude of 20%) compared to maceration. Increasing the ultrasound amplitude to above 20% (30, 40, 50, and 60%) increases tryptophan fluorescence intensity; 17.550, 18.628, 20.162, and 21.688, respectively. This implies that ultrasound promotes pumpkin leaf protein folding, resulting in decreased exposure of chromogenic groups (i.e., hydrophobic groups) to the polar environment and subsequent interaction with the ANS probe (decreasing the *H*_0_ values, Figure 4A), eventually culminating in enhanced tryptophan fluorescence intensity. The tryptophan fluorescence intensity at 70% amplitude is lower than that of the other amplitudes (16.342), due to the stronger cavitation effect caused by unfolding the pumpkin leaf protein, disrupting the hydrophobic interaction of the protein, and exposing hydrophobic groups or regions outside the molecular chain, resulting in an enhanced *H*_0_ value.

According to the published research, studies on the topic of protein extraction from green leaves employing ultrasound are practically absent; hence, there are few results on the effect of ultrasound on structural changes in leaf proteins. Only three different ultrasound procedures were utilized: pulsed ultrasound-assisted extraction (60% amplitude) was used to isolate protein concentrate from sugar maple leaf [33], ultrasound probe treatment (25 kHz, power density 450 W/L) was used to isolate mulberry leaf protein [24], and an ultrasonic bath (42 kHz) was used to modify jackfruit leaf proteins [34]. These three studies’ findings demonstrate that distinct ultrasound amplitudes affect protein molecules’ structures along with their functional properties, and it is noteworthy how closely these findings align with the findings of our own study.

We chose ATR-FTIR spectroscopy to evaluate the secondary structure changes in the collected RuBisCO-rich protein fraction. Pumpkin leaf protein absorption bands (Supplementary Materials: Figure S2) are divided into three regions: amide I (1600–1700 cm^−1^), amide II (1530–1550 cm^−1^), and amide III (1260–1300 cm^−1^). The amide I band peak is attributed to the C=O stretching vibration of the amide group. The amide II band absorption is mainly due to the deformation and stretching vibrations of the N–H and C–N groups [35]. In ultrasound versus maceration, the protein structure was changed by the cavitation effect, as demonstrated by the shifting of the amide band in the spectra of protein fractions isolated from pumpkin leaves after ultrasound treatment at amplitudes ranging from 20 to 70%. The results showed that ultrasound treatment raised the absorbance of pumpkin leaf proteins (at 3300 cm^−1^) by three times compared to maceration, as well as resulting in a shift to a higher wavelength. This revealed that applying ultrasound amplitudes improved the protein’s intramolecular or intermolecular hydrogen bonding due to shear forces under the cavitation effect.

Furthermore, the secondary structures were estimated from the sum of the relative area of the peaks from amide I centered at the following absorption frequencies: intermolecular *β*-sheet (1610–1627 cm^−1^), *β*-sheet (1628–1642 cm^−1^), random coil (1643–1650 cm^−1^), *α*-helix and Gln sidechain (1650–1659 cm^−1^), and *β*-turn (1660–1699 cm^−1^) [35], and the data are listed in Table 2. The analysis of the amide I spectral area reveals that the protein spectra obtained through the maceration process are noticeably weaker than the protein fractions’ spectra obtained through ultrasound (Figure 4C). It is evident that the applied ultrasound amplitude affects the overall spectrum’s intensity of amide I. The spectrum expands when the amplitude increases from 20 to 40%, but it reduces when the amplitude increases even further to 50, 60, and 70%. The alterations in the spectrum between maceration and ultrasound previously described are confirmed based on the percentage yield of the proteins’ secondary structure (Table 2). When high-intensity ultrasound is applied instead of maceration, the proportion of *α*-helix protein structure decreases (from 36.71 to 12.29%), but the proportion of *β*-turn and *β*-sheet extended structures increases (from 13.64 to 30.97%, and from 15.09% to 23.57%, respectively). The described changes tell us that, in relation to maceration, ultrasound led to the unfolding of protein molecules and greater exposure of hydrophobic regions. Likewise, the mentioned changes are consistent with the surface hydrophobicity data that were described (Figure 4A), since the ultrasound sample (20% amplitude, 20 kHz) has a significantly greater amount of *H*_0_ than the maceration sample.

When it comes to ultrasound extraction as a treatment, we can point out that by increasing the ultrasound amplitude in the range of 20 to 50%, random coil and *β*-turn secondary structures remain very stable and are unchanged. Vice versa, the protein’s *α*-helix structure gradually grows from 20 to 70% of the amplitude, whilst the *β*-sheet (extended and intermolecular) diminishes with an irregular dependence on the applied amplitude. It can be emphasized that the optimal cavitation breaks down the pumpkin leaf protein’s hydrogen bonds, resulting in fewer *α*-helices and conversion to *β*-sheets and *β*-turns. On the other hand, excessive cavitation caused pumpkin leaf protein molecules to aggregate into clusters (amplitudes 60 and 70%). The mentioned observations are partially supported by the results of Zhao et al. [24] who indicated that ultrasound enhanced the content of *β*-sheet and random coil of mulberry leaf proteins by 11% and 15%, whilst it reduced *α*-helix and *β*-turn by 8 and 6%, respectively. 

#### 2.2.3. Scanning Electron Microscopy Analysis

**Figure 5:**
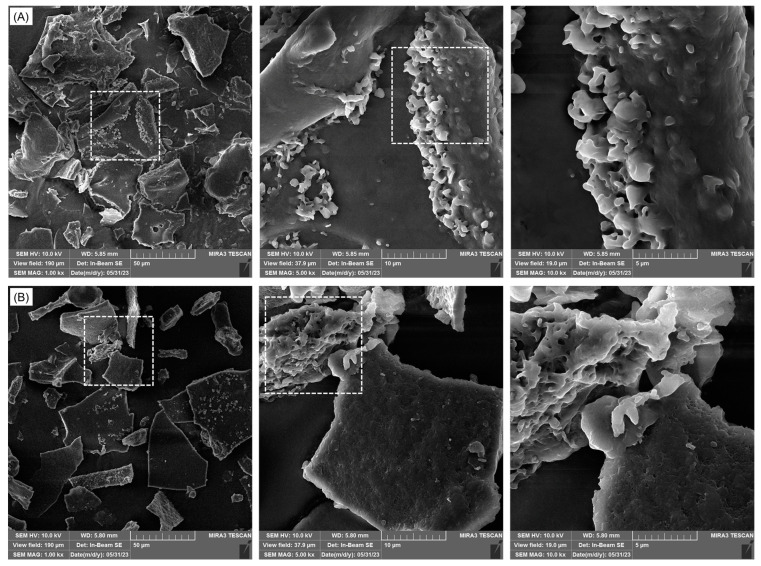
FESEM images (magnification 1000, 5000, and 10,000× from left to right) of RuBisCO-rich proteins extracted from pumpkin leaves by maceration (**A**) and high-intensity ultrasound with an amplitude of 40% (**B**).

The morphological changes induced by different extraction methods, namely maceration and ultrasound-assisted extraction (optimal condition for the abovementioned structural changes: 10 min, 40% amplitude), were visualized by FESEM images for RuBisCO-rich protein fractions (Figure 5). In summary, the surface of pumpkin protein particles differed in terms of structure and particle size. Firstly, the pumpkin leaf concentrates generated by both extraction processes had irregular and flake-like forms without spherically shaped (globular) protein bodies. This could be attributed to the solubilization of the fraction at pH 7 after precipitation and subsequent freeze-drying. When protein samples were extracted using ultrasound technology, their surfaces were smoother, and their structures were less organized. The cause of this alteration is the cavitation bubbles that are created during ultrasonic vibration, which have the impact of decaying plant cells. When protein extraction was performed with the maceration protocol, the texture became dispersive, and it exhibited more irregular fragments with disordered arrangements over the ultrasound extraction protocol. Likewise, in both DLS (dynamic light scattering) and FESEM measurements, the measured particle sizes of the extracted protein fractions coincided (260–390 nm). 

### 2.3. Functionality of RuBisCO-Rich Protein Fractions

**Figure 6:**
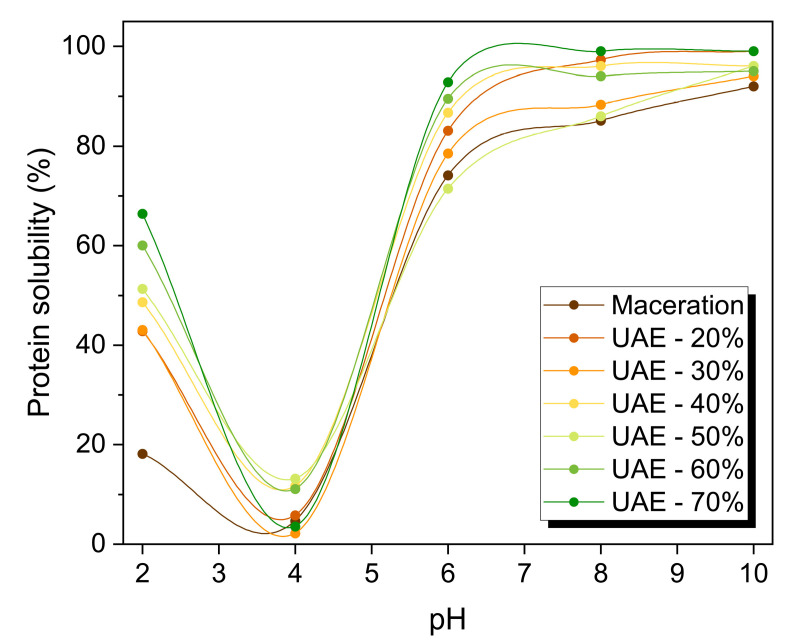
The solubility profile of the RuBisCO-rich protein fractions from pumpkin leaves exposed to different pH values. Legend: UAE-20%, UAE-30%, UAE-40%, UAE-50%, UAE-60%, and UAE-70% mean ultrasound-assisted extraction process at 20, 30, 40, 50, 60, and 70% amplitudes, respectively.

Protein–water interactions influence the functionality of primary constituents, such as emulsions, foams, and gels. The interaction of oil and water with proteins is important in food processing, since it modifies the textures and flavors of foods [14]. In this regard, the protein solubility, water and oil-holding capabilities, and emulsifying properties of isolated RuBisCO-rich protein fractions were investigated, and the results are graphically depicted in Figure 6 and tabulated in Table 3. When employing the high-intensity ultrasound, the functional characteristics of the isolated Rubisco-rich protein fraction differ considerably (*p* < 0.05) from those of the protein after maceration.

The solubility of pumpkin leaf proteins increased significantly (*p* < 0.05) with various ultrasound amplitudes at pH 2 and 4. At pH 2, soluble protein in the maceration sample had a solubility of 18.2 ± 1.10%. With ultrasound, an upward trend within the solubility of 2.4-fold (20% amplitude; 43.1 ± 1.25%) is immediately apparent, subsequent to an enormous increase in solubility as the amplitude grows higher (30 to 70%). Ultrasound treatment (70% amplitude) increased solubility up to 66.4 ± 3.30%, with a 3.6-fold increase. We additionally observed a remarkable increase in solubility within the pH range of 4–6, including evident increments of up to 20%. The increased solubility of ultrasound-treated RuBisCO-rich protein fractions was attributed to the loss of protein conformation, where acoustic cavitation and intense vibrations of ultrasound could induce some physical and/or chemical modifications in solutions through cavity breakdown and cyclic generation [20,26]. These phenomena caused turbulent flow and pressure in the immediate area of these spaces, increasing solubility (by modifying noncovalent interactions, such as Van der Waals forces, electrostatic drive, and hydrogen bridges) and converting insoluble protein into soluble ones. Ultrasonic technology may have affected the three-dimensional structure of globular proteins, resulting in increased numbers of charged clusters and improved electrostatic forces [18,22,26]. Consequently, more protein molecules interacted with water molecules, increasing the solubility.

In comparison to maceration, the emulsifying ability decreases; increasing the ultrasound amplitude from 20 to 60% reduces the measured value of *EAI* by 24.5–41.5% (Table 3), which is positively correlated with the above-described behavior of surface hydrophobicity; reducing the surface hydrophobicity of proteins from pumpkin leaves results in a reduction in the emulsifying activity index. The stability of the emulsions generated diminishes with increasing ultrasonic amplitude, showing that 20% (20 kHz, 10 min) is adequate for isolating pumpkin leaf protein concentrate with appropriate functionality. Added emulsifying capabilities can be increased through a pH-shifting technique or the wake heat treatment, which results in a better balance of hydrophobic and hydrophilic residues capable of stabilizing the oil–water interface. At a 20% amplitude, *WHC* and *OHC* are not statistically different from maceration (Table 3; *p* > 0.05), indicating that UAE did not impair protein functionality, while causing major structural alterations. Higher amplitudes of 20% produce a drop in *WHC* and *OHC* in isolated proteins, which can be attributed to intramolecular and electrostatic interactions between structurally altered protein molecules. This reduction in *WHC*/*OHC* values is attributed to the denaturation of isolated pumpkin leaf proteins’ structures following cavitation (especially at higher amplitudes than 40%).

Earlier studies found that *WHC*, *OWC*, and emulsifying properties improved after cavitation [17,24,25,26]. This contradicts our findings due to the complexity of the pumpkin leaf as a raw material for protein extraction (cavitation affected the plant cells and a set of all of the macromolecules), as well as the fact that in most cases, only protein concentrate or isolate was used for ultrasound treatment. As such, further analyses are necessary to ascertain the influence of ultrasound on the pumpkin leaf protein extracts in terms of their emulsifying properties. 

## 3. Materials and Methods

### 3.1. Plant Material

The fresh pumpkin leaves (harvested September 2022) were obtained from a local pumpkin farm (*Cucurbita pepo* var. *oleifera*) owned by the company JS&O d.o.o. (Novo Milosevo, Serbia). To facilitate protein extraction, the leaves were first lyophilized (Beta 1–8 LSCbasic Freeze Dryer, Martin Christ GmbH, Osterode am Harz, Germany) and then ground into a rougher powder by using a mechanical ball mill (Mixer Mill MM400, Retsch, Germany) with a 2 × 12 mm stainless steel ball for 60 s at 25 Hz. The sample drying was performed under the following conditions: primary drying at −40 °C (pressure 0.12 mbar) for 23 h, and final drying for 1 h at −60 °C (pressure 0.011 mbar).

For quantitative determination of the proximate composition of lyophilized pumpkin leaves, the AOAC protocols were used [36]. Based on this compositional analysis, the freeze-dried pumpkin leaves have 27.53 ± 1.38% carbohydrate, 27.73 ± 1.39% protein, 22.61 ± 1.13% crude fiber, 11.31 ± 0.56% ash, 2.71 ± 0.14% crude fat, and 8.09 ± 0.40% moisture content.

### 3.2. Chemical Reagents

The chemical reagents that were required for spectrometric or fluorometric analysis of the RuBisCO samples were 8-anilino-1-naphthalene (ANS), sodium dodecyl sulphate (SDS), and bovine serum albumin (BSA), and these were purchased from Sigma Aldrich Co. (St. Louis, MO, USA). The bioactivities of RuBisCO-rich protein fractions have been tested using the following chemicals: 2,2′-azino-bis(3-ethylbenzothiazoline-6-sulfonic acid)-diammonium salt, ABTS, 2-thiobarbituric acid (Alfa Aesar, MA, USA), iron(II) chloride, 3-(2-pyridyl)-5,6-diphenyl-1,2,4-triazine-4′,4″-disulfonic acid (Ferrozine), 6-hydroxy-2,5,7,8-tetramethylchromane-2-carboxylic acid (Trolox), α-deoxyribose or 2-Deoxy-D-ribose, potassium persulfate and trichloroacetic acid (Sigma Aldrich, St. Louis, MO, USA), ethylenediaminetetraacetic acid disodium salt dihydrate (Tokyo Chemical Industry UK ltd., Oxford, UK), and hydrogen-peroxide (30%; Zorka Pharma-Hemija d.o.o, Šabac, Serbia). All other chemical reagents used were of analytical grade. The deionized water (18.2 MΩ) was generated using a Milli-Q purification system (Merck Millipore Advantage A10, Darmstadt, Germany).

### 3.3. Ultrasound-Assisted Extraction of Proteins from Pumpkin Leaves

The ultrasound-assisted extraction of proteins from pumpkin leaves with deionized water as a green extraction solvent was performed using ultrasound apparatus consisting of a Sonopuls Ultrasonic Homogeniser and a VS 70 T sonotrode-type probe (series HD 2200; Bandelin Electronic GmbH & Co. KG, Berlin, Germany). The ultrasonic homogenizer that was used has the following characteristics: an ultrasonic frequency of 20 ± 0.5 kHz and an input power of 30.70 ± 1.45 W. The diameter of the VS 70 T probe was 13 mm. Variations in the duration period (0, 1, 3, 5, 7.5, 10, 12.5, 15, 17.5, and 20 min) and amplitude (20, 30, 40, 50, 60, and 70%) were examined.

Protein extraction from pumpkin leaves was carried out with a constant solid-to-solvent ratio of 1:50 (*w*/*v*). The pumpkin leaf powder was resuspended in cold deionized water in a laboratory glass water jacket of 250 mL capacity and incubated for 30 min at 4 °C to prevent the activation of protease and polyphenol-oxidase. Then, a VS 70 T probe was immersed in the center of the beaker and the sample dispersion at a depth of about 2 cm, and the dispersion was irradiated with an ultrasound directly. Individual sample extraction temperatures were kept below 35 °C to avoid the denaturation of thermolabile proteins located in pumpkin leaves, and this was ensured by using glass water jackets filled with cold water and a certain amount of small ice cubes.

Following sonication, the sample dispersions were filtered through fast-flow filter paper using a Büchner funnel, and the liquid fraction was subjected to the thermocoagulation process in a 50 °C water bath. Denatured green membrane proteins were separated from the soluble protein fraction using a high-speed benchtop refrigerated laboratory centrifuge at 7830 rpm for 10 min (Eppendorf 5430 R; Eppendorf, Germany). Then, the sediment was discarded, and the supernatant was adjusted to pH 4.50 until water-soluble proteins were precipitated; protein precipitation was performed overnight at 4 °C. The RuBisCO-rich protein fraction as precipitate was separated by a centrifuge at 7830 rpm for 20 min; precipitates were washed twice with water, simultaneously adjusting the pH to 7, and subjected to subsequent centrifugation, after which the solid precipitate was freeze-dried. For comparison’s sake, the conventional extraction (maceration) was carried out as a control at ambient temperature under magnetic stirring at a speed of 200 rpm with a duration of 120 min. Following that, the maceration sample underwent the same steps as previously mentioned for the separation of the water-soluble protein fraction.

The fractions from the whole process phase were collected and utilized for subsequent analyses. The dry matter contents in the RuBisCO-rich fractions (solid and liquid streams) were determined on a moisture analyzer (Kern MLS-A, Balingen, Germany).

#### 3.3.1. Evaluation of Protein Content and Protein Recovery

The estimation of protein content was based on the colorimetric reaction between copper ions and side chains of tyrosine, tryptophan, and cysteine proteins in an alkaline environment. Regarding this, the procedure was performed according to the modified Lowry protocol [37]. For analysis, the RuBisCO-rich protein fractions were resuspended in the water and, if there was a need, centrifuged (Eppendorf MiniSpin, Eppendorf, Hamburg, Germany) for 10 min at 7800 rpm. The determination of the protein concentration was conducted by creating a calibration curve with BSA as the standard. The obtained concentration of total proteins as the mean value of the three measurements was finally expressed as protein recovery (the mass of extracted proteins) with respect to the dry matter content of the dried pumpkin leaf sample (mg/g DM).

In addition, during the study of the kinetics of protein extraction, the soluble protein extraction yield was measured as follows:

(2) $$Y\;\left(\%\right)=\frac{m_{ex}}{m_{0}^{\prime }}\cdot 100=\frac{m_{t}-m_{o}}{m_{0}^{\prime }}\cdot 100$$

where *Y* (%) denotes the soluble protein extraction yield; *m_ex_* (mg) denotes the mass of soluble proteins; *m_t_* and *m_o_* (mg) denote the mass of soluble proteins at the extraction time *t* and at the beginning (*t* = 0 min), respectively; and *m*_0_^′^ (mg) denotes protein content in the raw leaf sample.

#### 3.3.2. Evaluation of Total Polyphenol Content and Polyphenol Recovery

The evaluation of total phenolic compounds was based on colorimetric reactions between Folin–Ciocalteu reagent and the phenolic compounds [38]. The RuBisCO-rich sample aliquots of 0.05 mL, 3.7 mL of deionized water, 0.25 mL of 2 M commercial Folin-Ciocalteu reagent, and 1 mL of a 15% (*w*/*w*) sodium carbonate solution were added, thoroughly mixed, and left to stand in the dark for two hours. The absorbance change in the samples relative to the blank (0.05 mL of deionized water instead of protein samples) was measured utilizing a UV–Vis spectrophotometer (Ultrospec 3300 pro, Amersham Bioscience, Slough, UK) at 750 nm.

The polyphenol recovery as the mean of the three measurements was expressed as equivalents of gallic acid (GAE) with respect to the dry matter content of the lyophilized pumpkin leaves (mg GAE/g DM).

### 3.4. Molecular Structure and Morphology Characterization of RuBisCO-Rich Protein Fractions

#### 3.4.1. Evaluation of the Presence of RuBisCO Protein Subunits

SDS-PAGE analysis according to Laemmli’s protocol [39] was used to evaluate the efficiency of high-intensity ultrasound for the isolation of the RuBisCO protein fraction. Briefly, electrophoresis was performed by using a polyacrylamide gel with a concentration of 8% (mPAGE™ 8% Bis-Tris Precast Gel; 10 × 8 cm, 12-wells; Merck KGaA, Darmstadt, Germany) on a mini vertical electrophoresis system (SE260 Mighty Small II Deluxe Mini Vertical Protein Electrophoresis Unit; Hoefer Inc., Holliston, MA, USA). Prior to use, the soluble protein samples (10 mg/mL) were mixed in a volume ratio of 1:1 with sample buffer (2.5 mL of Tris-HCl buffer, 2.0 mL of glycerol, 2.0 mL of 1M DTT solution, 4.0 mL of 10% SDS, and 0.4 mL of 5% bromophenol blue) and heated at 95 °C for 5 min. Prepared samples (4 µL) were loaded onto gel along with pre-strained protein markers (10–260 kDa; Spectra Multicolor Broad Range Protein Ladder; Thermo Scientific, Waltham, MA, USA). The separation was performed with commercial running buffer (MES SDS running buffer powder; Merck KGaA, Darmstadt, Germany) under 120 V current for 50 min. Coomassie Brilliant Blue R-250 was used to tint the gel (10% acetic acid, 45% methanol, and 0.25% CBB R-250). Then, the decolorization of the gel was performed over two hours using a solution with 10% *v*/*v* acetic acid and 45% *v*/*v* methanol with a couple of changes and a 30 min incubation. 

#### 3.4.2. Evaluation of Intrinsic Fluorescence Spectra and Surface Hydrophobicity

The fluorescence ANS probe was applied to measure surface hydrophobicity [40]. Leaf RuBisCO-rich protein dispersions (0.5% *w*/*w*) were diluted with phosphate buffer (0.01 M, pH 7.0) to a range of protein concentrations (0.005 to 0.04% (*v*/*v*)). A total of 7 μL of ANS was added to 1.393 mL protein solutions at different concentrations. The mixture was incubated for 15 min while protected from light. Fluorescence intensity was read at 25 °C using a Horiba FluoroMax-4 spectrofluorometer (Horiba Jobin Yvon GmbH, Oberursel, Germany) at 360 nm (excitation) and 480 nm (emission) with a constant excitation and emission slit of 2.5 nm and 10 nm/s of scanning speed. Surface hydrophobicity was expressed as the initial slope of the plot of normalized relative fluorescence intensity (relative to the ANS probe) as a function of protein concentration by using linear regression analysis.

The fluorescence spectra of tryptophan were measured based on Li et al. [41]. The protein samples were thinned to 0.1% (*w*/*w*) with a phosphate buffer (0.01 M, pH 7.0). The 2 mL of dispersions were placed in a quartz cell. The spectrum was obtained with a scanning speed of 40 nm/s at the excitation and emission wavelengths of 280 nm and 300–500 nm, respectively, using the aforementioned spectrofluorometer.

#### 3.4.3. Secondary Protein Structure Analysis by Used FTIR Spectrometry

The spectra of leaf RuBisCO-rich proteins were acquired in transmission mode using a Nicolet™ iS™10 FT-IR spectrometer (Thermo Fisher Scientific, Madison, WI, USA) with Smart iTR™ attenuated total reflectance (ATR) sampling accessories. ATR-FTIR spectra (20 accumulated scans at 4 cm^−1^ resolution) were collected over the frequency range of 500 to 4000 cm^−1^. The spectra of leaf protein samples were performed in triplicate and the results were reported as the averages of these replicates (relative standard deviation < 5%). The secondary leaf protein structures were investigated in the amide I region (1600–1700 cm). Deconvolution was performed and analyzed by using the OriginPro 19.0 software (Fit Peaks Pro) through baseline subtraction, deconvolution, second derivative with the Savitzky–Golay function, and peak fitting using the Gaussian function.

#### 3.4.4. Evaluation of Average Diameter Size and Surface Electrical Charge

Using pure deionized water, leaf RuBisCO-rich protein dispersions (0.5% *w*/*w*) were first diluted 50-fold. The average particle size (*d*, nm), distribution of particle sizes by intensity and the polydispersity index (PdI) of the prepared leaf protein dispersion particles, were measured at 23 ± 0.5 °C, respectively, by dynamic light scattering and laser Doppler velocimetry using a Zetasizer Nano ZS device associated with the Mastersizer 2000 software package (version 6.12; Malvern Instruments Ltd., Malvern, UK). Deionized water was used as a dispersant, and the rheological values characteristic of this dispersion system were as follows: a diffraction index of 1.354 and index absorptivity of 0.001. The surface electrical charge of leaf protein dispersion, zeta potential (mV), was evaluated on the same device, by an electrophoretic mobility technique and by monitoring the particle’s motion in the electric field. The strength of the applied electric field was 20 V/cm. The dielectric constant was 65 at 23 ± 0.5 °C (300–450 MHz), and the viscosity was 10 mPa∙s. 

#### 3.4.5. Morphological Characterization by Used FESEM Analysis

Additionally, field-emission scanning electron microscopy (Tescan MIRA 3 XMU, Brno, Czech Republic) was used to examine the surface morphology of the freeze-dried RuBisCO-rich proteins. In order to prevent electrostatic charge, the protein powders were coated with Au in an argon atmosphere using a spatter coater (Polaron SC502, Thermo VG Microtech, West Sussex, UK) before being subjected to an FESEM analysis. Digital images were obtained at an accelerating voltage of 10 kV.

### 3.5. In Vitro Bioactivities of RuBisCO-Rich Protein Fractions

The antioxidant qualities of all samples have been evaluated with the objective of understanding the impact of high-intensity ultrasound on the biological activity of the RuBisCO-rich protein fractions. For the subsequent procedures, freeze-dried protein samples whose protein content was previously established have been used.

#### 3.5.1. Evaluation of ABTS^•+^ Radical Scavenging Activity

The ABTS^•+^ radical scavenging activity of extracted leaf protein fractions was determined using the procedure described by Knežević et al. [42]. The radical cation was generated by reacting the 7 mM ABTS with 140 mM potassium persulfate (so that a final concentration of 2.45 mM was achieved), and then the solution was left in the dark at room temperature for 12–16 h. The radical cation solution was diluted with 5 mM PBS buffer (pH 7.4) to an absorbance of 0.70 ± 0.02 at 734 nm before testing. An aliquot (10 μL) of each protein dispersion (concentrations from 0.01 to 0.5% (*w*/*w*)) was mixed with 0.320 mL of radical cation solution. The antioxidant activity was quantified by measuring the absorbance rate at 734 nm (microplate reader Multiscan GO, Thermo Scientific, Waltham, MA, USA), after 5 min of incubation in the dark. The degree of scavenging activity expressed as a percentage was calculated as the ratio of the reacted amount to the total amount of radical cation, as follows:

(3) $$Scavenging\;degree\;\left(\%\right)=\frac{{ABTS}_{reacted}}{{ABTS}_{total}}\cdot 100=\left(1-\frac{A_{sample}}{A_{control}}\right)\cdot 100$$

where *A_control_* and *A_sample_* represent the reading absorbances of control and sample, respectively. A calibration curve was plotted by reacting aqueous Trolox solution (0–0.8 mM) with diluted radical solution. Accordingly, the results were presented as Trolox-equivalent antioxidant activity (mmol TE/g protein).

#### 3.5.2. Evaluation of Ferrous Ions’ Chelating Activity

The ferrous ions’ chelating activity of extracted leaf protein fractions was measured using an approach published by Knežević et al. [42]. Briefly, the samples were made by mixing 0.05 mL of each protein dispersion (concentrations from 0.01 to 0.5% (*w*/*w*)) with 0.2 mL of deionized water. After that, 0.025 mL of a 2 mM reagent solution of iron dichloride was added, and the solution was intensively vortexed and incubated for 3 min at 20 °C. Then, the reaction was initiated by the addition 0.05 mL of 5 mM ferrozine and the mixture was incubated at 20 °C for 10 min after rigorous mixing. Deionized water was used to create the control sample as the substitute of a protein sample, and a comparable process was used to create the blank sample, wherein deionized water was used in lieu of the reagents. The ferrous chelating activity was quantified using microplate reader spectrophotometer (Multiscan GO, Thermo Scientific, Waltham, MA, USA) by measuring the ferrous ferrozine complex’s change in absorbance at 562 nm. The degree of chelating activity was calculated as the amount of the reacted over the total amount of ferrous ions, as follows:

(4) $$Chelating\;degree\;\left(\%\right)=\frac{{Fe}_{reacted}^{2+}}{{Fe}_{total}^{2+}}\cdot 100=\left(1-\frac{A_{sample}-A_{blank}}{A_{control}}\right)\cdot 100$$

where *A_control_*, *A_sample_*, and *A_blank_* represent the reading absorbances of the control, sample, and blank, respectively. A calibration curve was plotted by reacting aqueous Na_2_EDTA (0–0.3 mM) with the reactants of the above-described protocol. The capacity of each extracted protein samples to chelate ferrous ions was presented as EDTA equivalent antioxidant activity (mmol EE/g protein).

#### 3.5.3. Evaluation of Hydroxyl Radical Scavenging Activity

The hydroxyl radical scavenging activity of extracted leaf protein fractions was assessed using the *α*-deoxyribose oxidation protocol reported by Chung et al. [43] with minor modifications. An aliquot of 0.25 mL of each protein dispersion (concentrations from 0.01 to 0.5% (*w*/*w*)) was mixed with 0.2 mL of 10 mM FeSO_4_-EDTA solution and 0.25 mL of 10 mM *α*-deoxyribose solution, and then the reaction mixture was supplemented with 0.1 M phosphate buffer (pH 7.4) to a volume of 0.9 mL. The Fenton reaction used to generate the hydroxyl radical was initiated by the addition of 0.1 mL 100 mM H_2_O_2_ mM, and the solution was then shaken vigorously and incubated in a water bath at 37 °C for 1 h. The control sample was prepared using the same procedure, but instead of a protein sample, it included deionized water, and the blank sample used deionized water in place of the reagents. After incubation, by pouring 0.5 mL of ice-cold 2.8% (*w*/*w*) trichloroacetic acid, the reaction was stopped, and by introducing 0.5 mL of 1% (*w*/*w*) thiobarbituric acid, the color was developed. Then, the mixture was allowed to stand at 90 °C for 15 min, and the absorbance at 532 nm was recorded (Ultrospec 3300 Pro, Amersham Bioscience, Slough, UK) to determine the hydroxyl radical scavenging activity as an inhibition rate of *α*-deoxyribose oxidation by hydroxyl radicals, as follows:

(5) $$Inhibition\;degree\left(\%\right)=\frac{{OH}_{reacted}^{•}}{{OH}_{total}^{•}}\cdot 100=\left(1-\frac{A_{sample}-A_{blank}}{A_{control}}\right)\cdot 100$$

where *A_control_*, *A_sample_*, and *A_blank_* represent the reading absorbances of the control, sample, and blank, respectively. A calibration curve was plotted by reacting aqueous Trolox (0–3 mM) with the reactants of the above-described protocol. The capacity of each extracted protein samples for scavenging hydroxyl radicals was presented as Trolox-equivalent antioxidant activity (mmol TE/g protein).

### 3.6. Functional Properties of RuBisCO-Rich Protein Fractions

The functional properties have been evaluated with the objective of understanding the impact of high-intensity ultrasound on the solubility, holding capacities, and emulsifying properties of the RuBisCO-rich protein fractions. For the subsequent procedures, freeze-dried protein samples whose protein content was previously established have been used.

#### 3.6.1. Evaluation of Protein Solubility

The water solubility profiles of RuBisCO-rich proteins were determined by preparing 1% (*w*/*w*) sample dispersions, and the pH values of the dispersions were adjusted to 2, 4, 6, 8, and 10 by using 0.2 M NaOH or 0.2 M HCl solutions. The samples were incubated at 20 °C for 0.5 h under constant stirring (typically 250 rpm) by using a magnetic stirrer. Aliquots of the dispersions were transferred to Eppendorf tubes and centrifuged at 12,100× *g* for 5 min at 4 °C (Heraeus Fresco 21 Microcentrifuge, Thermo Scientific, Waltham, MA, USA). The protein contents in the supernatant and in the whole sample at various pHs were measured using the modified Lowry method [35]. Solubility was expressed as the percentage of soluble proteins remaining in the supernatant as compared to the initial sample.

#### 3.6.2. Evaluation of Water-Holding and Oil-Holding Capacities

The extracted RuBisCO-rich protein fractions’ ability to bind oil was measured in accordance with our earlier descriptions [44]. In a centrifuge tube, 0.25 g of powdered extracts were combined with 5 mL of sunflower oil. The mixture was then maintained at 20 °C for 30 min while being stirred every 10 min. After centrifuging the mixture for 20 min at 4000× *g* (Sigma 2-16P, Sigma, Osterode am Harz, Germany), the volume of the supernatants was measured and weighed. The amount of fat absorbed per gram of protein was calculated using the obtained weights. In a blank tube, fat adherence to the tube walls was calculated.

With a few minor adjustments, the isolated RuBisCO protein’s capacity to absorb water was established [44]. In a centrifuge tube, 0.5 g of protein powder was swirled with 5 mL of distilled water. After incubation at 20 °C for 30 min, the tubes were centrifuged for 20 min at 4000× *g* (Sigma 2-16P, Sigma, Osterode am Harz, Germany). The supernatant volume was weighed. The amount of water gripped by one gram of protein served as the unit of measurement for the water-holding capacity.

In both instances, the estimated total masses relied on for calculations included the total mass of the protein samples prior to centrifugation, the total mass of the centrifuge tube and sample prior to centrifugation, and the total mass of the centrifuge tube and sample post centrifugation.

#### 3.6.3. Evaluation of Emulsification Activity and Stability Index

Prepared RuBisCO-rich protein fractions were analyzed by the turbidimetric technique for emulsion activity index and emulsion stability index [45]. Emulsions (O/W) of each protein dispersion (1% *w*/*w*) were prepared with sunflower oil in a molar ratio of 3:1 (*v*/*v*) and mixed for 60 s with a laboratory homogenizer at 9500 rpm (Yellowline, DI 25 basic, IKA-Werke Gmbh & Co., Staufen, Germany). The absorbance of the diluted emulsions (a 20 µL aliquot of emulsion was diluted 100-fold with 0.05 phosphate buffer plus 0.1% *w*/*v* SDS, pH 7) was measured at 500 nm (Ultrospec 3300 Pro, Amersham Bioscience, Slough, UK). The turbidity was calculated by the following equation:

(6) T=2.303·l

where *T* is turbidity and *l* is a path length surface (cm^2^). The emulsion activity index (*EAI*) was then calculated as follows:

(7) $$EAI\;\left(\frac{m^{2}}{g}\right)=\frac{2\cdot T\cdot A_{0}\cdot d_{f}}{\theta \cdot c\cdot 10,000}$$

where *A* is absorbance; *θ* is the volume fraction of the dispersed phase—oil; *c* is the weight of protein per unit volume of aqueous phase before the emulsion is formed (g); and *d_f_* is the dilution factor and was calculated as being one hundred. For determining emulsion stability, the protein dispersions were kept at 10 min at 20 °C and analyzed for emulsion activity as previously described. An emulsion stability index (*ESI*) was calculated using the following formula:

(8) $$ESI\;(h)=\frac{A_{0}}{(A_{0}-A_{t})\cdot t}$$

where *t* represents the time interval observed for emulsion stability.

### 3.7. Statistical Analysis

Triplicate measurements were taken, and the final results were expressed as mean ± standard deviation (SD). The statistical analysis of the data was performed using OriginPro19.0 software (Origin Lab Corporation, Northampton, MA, USA). A comparison of the means was ascertained by Tukey’s test at 5% significance level using a one-way analysis of variance (ANOVA).

## 4. Conclusions

To the best of our knowledge, the study’s findings examine the impact of ultrasound on the structural and functional characteristics of RubisCO-rich protein fractions that have been isolated from green pumpkin leaves for the first time. The protein fractions that were isolated are distinctive, since they were extracted utilizing an UAE after the leaves were mechanically ground and lyophilized. Both RuBisCO protein subunits were detected as the most dominant protein subunits, and a couple of membrane protein subunits have been recognised from the other bands. With a protein content ranging from 57% to 63% along with a protein recovery of 110 mg/g DM, the isolated fractions were described as protein concentrates under ideal UAE conditions, which included a time of 10 min, amplitude of 40%, and frequency of 20 kHz. Cavitation caused the disruption of the pumpkin leaf wall, causing a large amount of water to penetrate into the cellular material, improve mass transfer, and release the cell content, which improves the protein extraction yield. When compared to maceration, UAE altered the leaf protein’s secondary and tertiary structures, causing the protein to unfold and exposing its hydrophobic groups. Smaller particle size, improved dispersion, and improved functional qualities, including enhanced solubility, water-holding capacity, and emulsifying stability—all essential characteristics of protein in food applications—were seen in the protein extracted by UAE.

## Figures and Tables

**Table 1:** Antioxidant activity as a measure of the bioactivity of the extracted leaf protein fractions.

Samples	ABTS Radical Scavenging Activity (mmol TE/g)	Metal-Ion Chelating Activity (mmol EE/g)	Hydroxyl Radical Scavenging Activity (mmol TE/g)	Protein Content (%)
Maceration	0.871 ± 0.058 *^ab^*	0.380 ± 0.022 *^c^*	1.380 ± 0.023 *^ac^*	52.07 ± 1.25 *^b^*
UAE-20%	0.893 ± 0.058 *^a^*	0.364 ± 0.026 *^c^*	1.540 ± 0.069 *^bc^*	54.52 ± 2.78 *^b^*
UAE-30%	0.794 ± 0.032 *^abc^*	0.545 ± 0.014 *^a^*	1.716 ± 0.025 *^b^*	62.47 ± 2.07 *^a^*
UAE-40%	0.657 ± 0.035 *^c^*	0.462 ± 0.014 *^b^*	2.126 ± 0.016 *^a^*	56.92 ± 0.34 *^b^*
UAE-50%	0.521 ± 0.032 *^d^*	0.248 ± 0.013 *^d^*	2.072 ± 0.032 *^a^*	63.95 ± 0.39 *^a^*
UAE-60%	0.443 ±0.029 *^e^*	0.229 ± 0.013 *^d^*	1.371 ± 0.015 *^c^*	63.06 ± 0.05 *^a^*
UAE-70%	0.436 ±0.025 *^e^*	0.225 ± 0.013 *^d^*	1.334 ± 0.015 *^a^*	62.51 ± 2.83 *^a^*

The data are presented as the mean ± SD (*n* = 3). Means with different letters for the same antioxidant activities of leaf protein (columns) are significantly different (*p* < 0.05). Legend: UAE-20%, UAE-30%, UAE-40%, UAE-50%, UAE-60%, and UAE-70% mean ultrasound-assisted extraction process at 20, 30, 40, 50, 60, and 70% amplitudes, respectively.

**Table 2:** Secondary structure band assignments in the RuBisCO-rich protein fractions obtained via ultrasound-assisted extraction from pumpkin leaves.

Secondary Structure	Band Assignment in the RuBisCO-Rich Protein Fraction (%)						
	Without Ultrasound *	UAE-20%	UAE-30%	UAE-40%	UAE-50%	UAE-60%	UAE-70%
*β*-sheet (intermolecular)	34.55 ± 1.09 *^a^*	19.58 ± 1.12 *^b^*	20.02 ± 1.00 *^b^*	15.08 ± 2.04 *^c^*	16.51 ± 1.55 *^c^*	16.33 ± 1.25 *^c^*	18.22 ± 1.30 *^b^*
*β*-sheet (extended)	15.09 ± 1.06 *^c^*	23.57 ± 1.15 *^a^*	18.95 ± 0.95 *^b^*	17.61 ± 1.24 *^b^*	20.14 ± 1.80 *^ba^*	22.19 ± 1.20 *^a^*	23.28 ± 1.45 *^a^*
Random coil	n.d.	13.60 ± 0.85 *^b^*	10.48 ± 1.45 *^b^*	20.39 ± 2.54 *^a^*	11.16 ± 2.02 *^b^*	5.21 ± 1.54 *^c^*	n.d.
*α*-helix	36.71 ± 1.14 *^b^*	12.29 ± 1.65 *^e^*	18.59 ± 1.42 *^d^*	20.44 ± 1.72 *^c^*	25.32 ± 2.00 *^c^*	37.67 ± 1.40 *^b^*	57.24 ± 1.20 *^a^*
*β*-turn	13.64 ± 1.24 *^d^*	30.97 ± 1.42 *^a^*	31.98 ± 2.01 *^a^*	26.49 ± 2.00 *^b^*	26.87 ± 1.72 *^b^*	18.61 ± 2.64 *^c^*	1.26 ± 0.25 *^e^*

* Maceration process performed under the same reaction conditions, but without ultrasonication. n.d.—not determined. The data are presented as the mean ± SD (*n* = 3). Means with different letters for the same secondary structure of proteins (row) are significantly different (*p* < 0.05). Legend: UAE-20%, UAE-30%, UAE-40%, UAE-50%, UAE-60%, and UAE-70% mean ultrasound-assisted extraction process at 20, 30, 40, 50, 60, and 70% amplitudes, respectively.

**Table 3:** Emulsifying properties (activity index and stability index) and water/oil-holding capacities of the RuBisCO-rich protein fractions extracted from pumpkin leaves.

Samples	*WHC* (g/g_proteins_)	*OHC* (g/g_proteins_)	*EAI* (m^2^/g_proteins_)	*ESI* (h)
Maceration	4.05 ± 0.58 *^ab^*	6.58 ± 0.51 *^a^*	8.77 ± 0.25 *^a^*	3.56 ± 0.058 *^ab^*
UAE-20%	4.71 ± 0.78 *^a^*	5.15 ± 0.42 *^a^*	6.62 ± 0.44 *^b^*	3.97 ± 0.065 *^a^*
UAE-30%	3.72 ± 0.62 *^b^*	3.94 ± 0.52 *^b^*	5.54 ± 0.24 *^c^*	3.34 ± 0.054 *^b^*
UAE-40%	2.43 ± 0.45 *^c^*	2.91 ± 1.07 *^b^*	5.21 ± 0.45 *^c^*	1.66 ± 0.073 *^c^*
UAE-50%	2.98 ± 0.62 *^c^*	3.15 ± 0.38 *^b^*	5.51 ± 0.31 *^c^*	1.11 ± 0.085 *^d^*
UAE-60%	2.48 ± 0.39 *^c^*	2.58 ± 0.34 *^c^*	5.13 ± 0.22 *^c^*	0.28 ± 0.076 *^e^*
UAE-70%	2.20 ± 0.35 *^c^*	2.18 ± 0.33 *^c^*	n.d.	n.d.

n.d.—not determined (reliable measurements at experimental conditions were not possible). The data are presented as the mean ± SD (*n* = 3). Means with different letters for the same protein functionality are significantly different (*p* < 0.05). Legend: UAE-20%, UAE-30%, UAE-40%, UAE-50%, UAE-60%, and UAE-70% mean ultrasound-assisted extraction process at 20, 30, 40, 50, 60, and 70% amplitudes, respectively.

## Data Availability

The data presented in this study are available from the corresponding author upon request.

## Supplementary Materials

The following supporting information can be downloaded at: https://www.mdpi.com/article/10.3390/molecules29174027/s1, Figure S1: Extraction kinetics of pumpkin leaf proteins during the maceration process. Figure S2: ATR-FTIR spectra of the RuBisCO-rich protein fractions isolated from pumpkin leaves via ultrasound emerging technology (frequency 20 kHz; various amplitudes) and maceration. Table S1: Free amino acids content in protein-rich samples obtained *via* ultrasound-assisted and maceration procedures determined by ninhydrin protocol.
