# Supplementary material for: Ultrasound as Green Technology for the Valorization of Pumpkin Leaves: Intensification of Protein Recovery
Source: Molecules. 2024 Aug 26;29(17):4027. doi: 10.3390/molecules29174027 (PMC11396713; doi:10.3390/molecules29174027)
Supplement: Supplementary file 1 [file molecules-29-04027-s001.zip › molecules-3142512-supplementary.pdf]

SUPPLEMENTARY MATERIAL

Ultrasound as Green Technology for the Valorization of Pumpkin Leaves: Intensification of Protein Recovery

Jelena Mijalković [1], Nataša Šekuljica [2], Sonja Jakovetić Tanasković [1], Predrag Petrović [2], Bojana Balanč [2], Marija Korićanac [1], Ana Conić [1], Jelena Bakrač [2], Verica Đorđević [3], Branko Bugarski [3] and Zorica Knežević-Jugović [1,*]

[1] Department of Biochemical Engineering and Biotechnology, Faculty of Technology and Metallurgy,
University of Belgrade, Karnegijeva 4, 11000 Belgrade, Serbia; jjovanovic@tmf.bg.ac.rs (J.M.); sjakovetic@tmf.bg.ac.rs (S.J.T.); mkoricanac@tmf.bg.ac.rs (M.K.); aconic.tmf@gmail.com (A.C.)
[2] Innovation Centre of the Faculty of Technology and Metallurgy Ltd., Karnegijeva 4, 11000 Belgrade, Serbia; nsekuljica@tmf.bg.ac.rs (N.Š.); ppetrovic@tmf.bg.ac.rs (P.P.); bisailovic@tmf.bg.ac.rs (B.B.); jbakrac@tmf.bg.ac.rs (J.B.)
[3] Department of Chemical Engineering, Faculty of Technology and Metallurgy, University of Belgrade,
Karnegijeva 4, 11000 Belgrade, Serbia; vmanojlovic@tmf.bg.ac.rs (V.Đ.); branko@tmf.bg.ac.rs (B.B.)
* Correspondence: zknez@tmf.bg.ac.rs; Tel.: +381-11-3303776

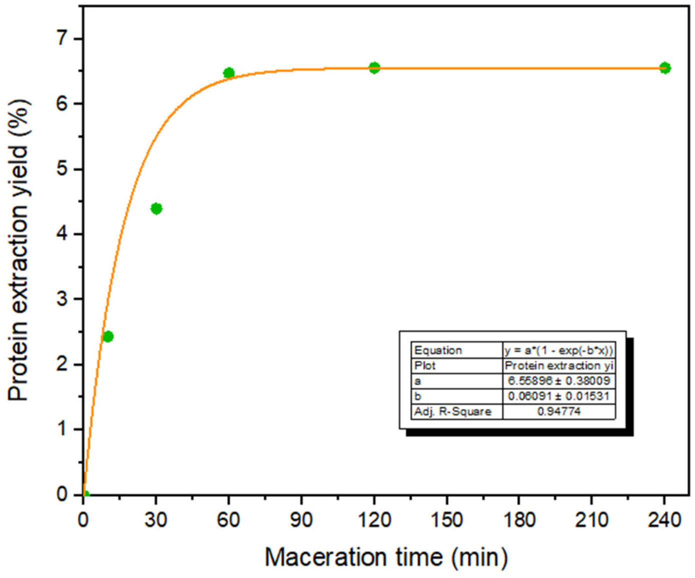

Figure S1: Extraction kinetics of pumpkin leaf proteins during maceration process.

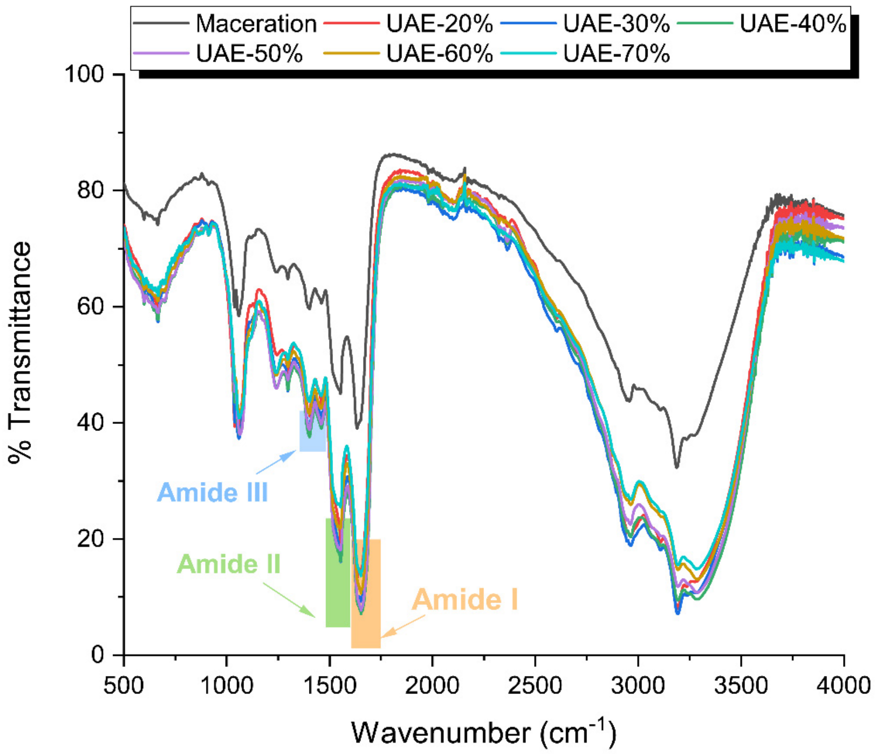

Figure S2: ATR-FTIR spectra of the RuBisCO-rich protein fractions isolated from pumpkin leaves *via* ultrasound-emerging technology (frequency 20 kHz; various amplitudes) and maceration.

Table S1: Free amino acids content in protein-rich samples obtained *via* ultrasound-assisted and maceration procedures determined by ninhydrin protocol.

Samples	Free amino acid content (meq amino acid/g of protein)	
	**Leu**	**Pro**
Maceration	0.387 ± 0.035 [b]	0.070 ± 0.035 [b]
UAE-20%	0.582 ± 0.040 [a]	0.278 ± 0.025 [a]
UAE-30%	0.601 ± 0.020 [a]	0.327 ± 0.031 [a]
UAE-40%	0.621 ± 0.029 [a]	0.304 ± 0.025 [a]
UAE-50%	0.614 ± 0.028 [a]	0.275 ± 0.036 [a]
UAE-60%	0.597 ± 0.036 [a]	0.250 ± 0.049 [a]
UAE-70%	0.590 ± 0.040 [a]	0.242 ± 0.046 [a]

Results are expressed as means ± standard deviation ($n = 3$).
Means with different letters for the same amino acid are significantly different ($p < 0.05$).
